# Pediatric Healthcare Worker Perspectives on Implementation of a Secure Firearm Storage Program: A Qualitative Study

**DOI:** 10.21203/rs.3.rs-7437500/v1

**Published:** 2025-10-08

**Authors:** Claire R. Waller, Mallika Pandey, Jennifer M. Boggs, Arne Beck, Ruth P. Bedoy, Alison M. Buttenheim, Marisa E. Elias, Katelin Hoskins, Shari Jager-Hyman, Christina Johnson, Melissa Maye, Bridget McArdle, Celeste Pappas, Dylan S. Small, LeeAnn M. Quintana, Courtney Benjamin Wolk, Leslie A. Wright, Nathaniel J. Williams, Brian K. Ahmedani, Rinad S. Beidas

**Affiliations:** Northwestern University; Northwestern Feinberg School of Medicine; Kaiser Permanente Colorado; Kaiser Permanente Colorado; Kaiser Permanente Colorado; University of Pennsylvania; Henry Ford Health; University of Pennsylvania; University of Pennsylvania; Northwestern University; Henry Ford Health; Henry Ford Health; Henry Ford Health; University of Pennsylvania; Kaiser Permanente Colorado; University of Pennsylvania; Kaiser Permanente Colorado; Boise State University; Henry Ford Health; Northwestern University

**Keywords:** implementation science, firearms, suicide prevention, injury prevention, pediatric primary care, well-child care

## Abstract

**Background:**

Primary care-based secure firearm storage programs are well-positioned to prevent firearm-related injury, the leading cause of death for young people in the United States. While recommended by the American Academy of Pediatrics and US Surgeon General, these programs have yet to become routine practice. Our cluster randomized hybrid effectiveness-implementation trial tested implementation strategies across 30 clinics in two large health systems for a universal evidence-based secure firearm storage program, *S.A.F.E. Firearm (Suicide and Accident Prevention through Family Education). S.A.F.E. Firearm* includes a brief discussion between a clinician and parent on secure firearm storage and an offer of free cable locks at pediatric well-child visits for youth ages 5–17. The ASPIRE trial demonstrated meaningful clinician behavior change, with *S.A.F.E. Firearm* delivered to a significantly higher percentage of patient families in the clinics that received both implementation strategies (49%) versus only one (22%). The present study qualitatively explores factors influencing the successful implementation of *S.A.F.E. Firearm*, centering healthcare worker (HCW) perspectives.

**Methods:**

Semi-structured qualitative interviews were conducted with leaders, clinic change agents, and clinicians involved in implementation from 2023–2024 (N = 38). The interview guide was informed by the original and updated Consolidated Framework for Implementation Research. Interviews were coded and analyzed using an abductive, integrated (i.e., deductive and inductive) approach. Inter-rater reliability (Kappa = 0.87) was strong.

**Results:**

Interviews elucidated four interconnecting themes. HCWs unanimously expressed pediatric HCWs’ responsibility to promote firearm safety (*role of pediatrics in firearm safety*) across heterogeneous *community and healthcare firearm cultures*. By preserving families’ *autonomy and privacy* around firearms, *S.A.F.E. Firearm*’s nonjudgemental and universal approach promoted program acceptability and delivery. Consequently, HCWs’ understanding versus *confusion around this universal, privacy-focused harm reduction approach* was foundational to implementation.

**Conclusions:**

Health systems can harness HCWs’ shared commitment to firearm safety by deploying brief programs that preserve recipient autonomy and privacy. To scale these evidence-based approaches, we recommend offering clear, simple trainings and collaboratively adapting programs to meet HCW and recipient needs.

**Trial registration:**

Registry: https://clinicaltrials.gov/study/NCT04844021, TRN: NCT04844021, first registered on April 14, 2021.

## Background

Firearm injury is the leading cause of death for young people in the United States (1). The American Academy of Pediatrics and US Surgeon General recommend implementation of secure firearm storage programs in pediatric primary care (2–4), given their potential to prevent firearm-related injury and mortality (2, 5–8). However, few pediatricians routinely discuss secure firearm storage with parents (28%) or offer firearm locks (2%), two key ingredients of these programs (6). A cluster randomized hybrid effectiveness-implementation trial (Adolescent and child Suicide Prevention in Routine clinical Encounters; “ASPIRE”) tested approaches to implement a brief universal evidence-based secure firearm storage program, *S.A.F.E. Firearm (Suicide and Accident Prevention through Family Education*), in pediatric primary care (8, 9). Thirty clinics across two health systems were randomly assigned to receive a new electronic health record documentation template (“nudge”) alone versus in combination with facilitation (interactive, clinic-level implementation support; “nudge+”). As previously reported (8), among 47307 well-child visits, a significantly higher percentage of young people and families received *S.A.F.E. Firearm* in nudge+ (49%) versus nudge (22%) clinics, demonstrating meaningful clinician behavior change that suggests the promise of taking this approach to scale.

*S.A.F.E. Firearm* (8, 9) includes a brief discussion between the pediatric clinician and parent on the importance of secure storage and an offer of free cable locks at well-child visits for youth ages 5–17. Our approach built upon adaptation of previous work (10) in partnership with end users (9, 11–13). Our adaptation centered a harm reduction approach, including nonjudgemental counseling that respects families’ autonomy and privacy regarding firearms; universally delivering the program irrespective of families’ firearm ownership status or storage practices; and including adolescents in the target population to better prevent both unintentional injury (the original focus) and suicide (our expansion in the adaptation process) (9, 11, 12). To inform scale-out of secure firearm storage programs and improve our implementation approach, we sought to contextualize ASPIRE trial findings in the experiences of healthcare workers (HCWs) involved in implementation (13). The present study qualitatively explores involved HCWs’ perspectives on the factors affecting implementation challenges and successes for *S.A.F.E. Firearm*.

## Methods

Semi-structured, qualitative interviews were conducted to understand *S.A.F.E. Firearm* implementation from the perspectives of HCWs (N = 38), including 8 leaders of pediatric primary care within the health systems, 10 clinic change agents (administrative staff and clinicians who were primary study point of contact within clinics), and 20 clinicians (pediatric and family medicine MDs, DOs, NPs, and PAs providing well-child care). Participants provided informed consent before interviewing and were recruited via email. The response rate was 31% overall (89% for leaders, 36% for clinic change agents, and 23% for clinicians). Of the 79 HCWs who did not participate, 91% did not respond to contact and 9% declined.

Leaders were selected to participate in qualitative interviews by the study team, which included embedded researchers within each health system. Clinic change agents were purposively sampled based on high versus low EHR-documented program delivery (reach) at their clinics: participants included 4 clinic change agents from high-reach clinics and 6 from low-reach clinics. Clinicians were purposively sampled to ensure variability across clinics, study arm (i.e., nudge versus nudge+), gender, race, and ethnicity. Clinicians reporting firearm ownership on a survey conducted before trial launch were oversampled. Self-reported gender (current gender identity), race, and ethnicity were assessed to capture participant representativeness.

The interview guide was informed by the original and updated Consolidated Framework for Implementation Research (see Supplement) (14, 15). Interviewers asked open-ended questions about participant experiences with program implementation and implementation strategies (i.e., EHR nudge, facilitation) as well as targeted follow-up probes. These questions queried both broad concepts (e.g., participant beliefs about firearm safety), and program-specific impressions (e.g., challenges to program deployment). The interview guide was pilot tested among the research team before deployment.

Interviewers (authors C.J., M.P., R.P.B.) and coders (authors C.R.W., M.P.) were female research staff with a master’s or bachelor’s level education. None had a significant prior relationship with any interviewees. Interviewers introduced their role when beginning interviews and took field notes during and immediately following interviews. Interviews were conducted virtually, audio-recorded, and lasted approximately 30 minutes. No one else was present for interviews, except for interviewers shadowing each other during training.

Interviews were conducted until data saturation was reached (2023–2024), professionally transcribed, and coded in NVivo 14. Coders reached and maintained excellent inter-rater reliability (Kappa = 0.87; 21% of transcripts double-coded). Coding and analysis employed an abductive and integrated approach, including both deductive and inductive methods (16–18). Prior to coding, the study team generated deductive codes, guided by the original and updated Consolidated Framework for Implementation Research (14, 15). Throughout coding, authors C.R.W. and M.P. generated and iteratively adjusted inductive codes. Analysis was guided by an abductive qualitative approach, which entails generating themes that best explain and synthesize patterns in deductive and inductive input (17–20). Interviewees did not review transcripts or results. COREQ guidelines were followed (21). The present paper describes qualitative findings on factors influencing program implementation and delivery; mixed-methods analyses specific to implementation strategy mechanisms are reported elsewhere (22).

## Results

Participant demographics are denoted in Table 1; participants predominantly identified as female and non-Hispanic White. We identified four interconnecting themes shaping program implementation: *role of pediatrics in firearm safety, community and healthcare firearm cultures; autonomy and privacy*, and *confusion around S.A.F.E. Firearm’s universal, privacy-focused harm reduction approach*. For all four themes, clinic change agent and leader input aligned with clinician reports on experiences implementing *S.A.F.E. Firearm*. We elaborate on how each theme shaped *S.A.F.E. Firearm* implementation below and share concrete examples of participants’ words in Table 2.

### Role of pediatrics in firearm safety

Participants unanimously expressed a belief that pediatric HCWs should leverage their roles to prevent firearm-related injury and mortality in youth, framing firearm safety as a public health issue. Some participants expressed that they viewed firearm safety discussions as a natural part of pediatric clinicians’ role akin to other universal, prevention-focused well-child visit topics, including sunscreen and seatbelts. Similarly, participants described how firearm safety is intrinsically linked with, and even embedded in, other key programs in pediatrics and healthcare broadly, such as suicide safety planning. Overall, participants expressed that they feel a responsibility to address firearm safety and are increasingly already broaching similar discussions in well-child visits, beyond programs like *S.A.F.E. Firearm*.

### Community and healthcare firearm cultures

While beliefs about the role of pediatrics in firearm safety were consistent across participants, there was significant variability in participants’ descriptions of community and healthcare firearm cultures, both within and across clinics. This theme includes beliefs about firearm ownership and safety among families, their local communities, and participants themselves. Most participants described heterogeneity in firearm ownership rates and firearm beliefs among families and the communities surrounding their clinics, noting polarization. They often stated that firearm-owning families varied in storage practices and reasons for ownership (e.g., hunting, personal safety in high-crime areas, law enforcement occupation). Some others described their patient families and local community as viewing firearms favorably, having high firearm ownership, and having a similar range in storage practices and reasons for ownership. No participants characterized their patient families or local community as primarily viewing firearms negatively or having low firearm ownership.

Participants described how the perceived prevalence of firearm ownership and unsecured firearms among families and in local communities drove them to prioritize *S.A.F.E. Firearm* delivery. Personal experiences with local firearm violence, including incidents directly affecting the well-being of patients and colleagues, reinforced participants’ belief that firearm injury prevention falls within the scope of pediatric care and promoted program delivery. Participants who disclosed personal experience with firearm ownership expressed that it gave them greater familiarity with and openness to firearm ownership compared to other HCWs. These participants shared that *S.A.F.E. Firearm* was inclusive of their views on firearm ownership because of its autonomy- and privacy-focused harm reduction approach.

### Autonomy and privacy

Many participants spontaneously emphasized their support for *S.A.F.E. Firearm*’s harm reduction approach, which prioritizes recipient autonomy regarding firearm ownership and a nonjudgmental manner. To promote recipient privacy, *S.A.F.E. Firearm* was designed to be universal: offered to all families without asking about or documenting their firearm ownership or storage practices. Some participants shared that this autonomy- and privacy-focused harm reduction approach enhanced *S.A.F.E. Firearm*’s acceptability for families. Participants highlighted that this approach increased families’ receptivity towards the program by being inclusive of different firearm ownership statuses and wide-ranging firearm beliefs, including both the belief that firearms can be dangerous for families and the belief in firearm ownership. Further, participants explained that by promoting families’ receptivity to the program, this inclusivity of families’ beliefs bolstered clinicians’ self-efficacy and made them more likely to deliver *S.A.F.E. Firearm*, particularly in the context of the politization of healthcare and medical mistrust.(23) Specifically, participants shared that HCWs were initially apprehensive about discussing firearms alongside other topics they viewed as “polarizing” and likely to elicit guardedness from families (e.g., COVID-19). However, these participants emphasized that delivering *S.A.F.E. Firearm* mitigated clinician concerns because the program’s approach made families more at ease. Many participants had previous experiences with programs that first screened families’ firearm ownership and storage and then offered targeted firearm safety discussions only to families who endorsed firearm ownership or unsecured firearm storage. These participants described *S.A.F.E. Firearm* as more effective than screening-based programs because it promoted recipient receptivity.

Participant input indicated that *S.A.F.E. Firearm*’s harm reduction emphasis and inclusivity of differing firearm beliefs not only made families more open to firearm discussions, but also fostered conversations around *S.A.F.E. Firearm* between HCWs. Participants described how, in the context of *S.A.F.E. Firearm* deployment, they had open exchanges of information with their colleagues of varied firearm cultures, enabling HCWs to support each other in learning to most effectively deliver *S.A.F.E. Firearm*. For example, some participants mentioned how some clinicians initially felt uncertain about delivering *S.A.F.E. Firearm* due to their own perceived lack of firearm knowledge. These participants described how colleagues at the clinic with more firearm knowledge helped clinicians to better understand how firearms and locks work, increasing clinicians’ self-efficacy to deliver *S.A.F.E. Firearm*. Similarly, participants discussed how familiarity with norms among firearm owners helped them to tailor *S.A.F.E*. Firearm for families, such as by acknowledging in discussion with parents that many firearm owners educate their children about firearm safety, but that there are still risks that secure firearm storage can help mitigate.

#### Confusion around S.A.F.E. Firearm’s universal, privacy-focused harm reduction approach

Given that the success of *S.A.F.E. Firearm* implementation was largely shaped by its autonomy- and privacy-focused harm reduction approach, participants’ full understanding of this approach was foundational to the program’s perceived acceptability and delivery. Some participants demonstrated a deep understanding of how to deliver *S.A.F.E. Firearm*. However, others believed they were meant to ask families about firearm ownership and/or storage practices, and then offer *S.A.F.E. Firearm* (i.e., firearm discussion, locks) only to families who screened positive for firearm ownership or unsecured firearm storage. Prior to *S.A.F.E. Firearm* implementation, many clinics had questionnaires that screened for firearm ownership and secure storage, which were not de-implemented upon *S.A.F.E. Firearm*’s launch.

Participant interviews revealed that this fostered confusion among HCWs, indicating the need to integrate programs like *S.A.F.E. Firearm* with pre-existing clinic processes to ensure synergy and avoid confusion that may be caused by seemingly conflicting initiatives. Further, participants’ input about how to avoid confusion illustrated how we may better promote HCWs’ understanding of programs like *S.A.F.E. Firearm* by leveraging targeted, succinct trainings and reminders that account for clinicians’ competing clinical priorities and high cognitive load. Participants additionally shared how creating space for clinicians, staff, and leaders to exchange learnings on program delivery, including leveraging audit and feedback, prevented confusion and solidified key takeaways about program delivery.

### Relationships between themes

**Figure 1:**
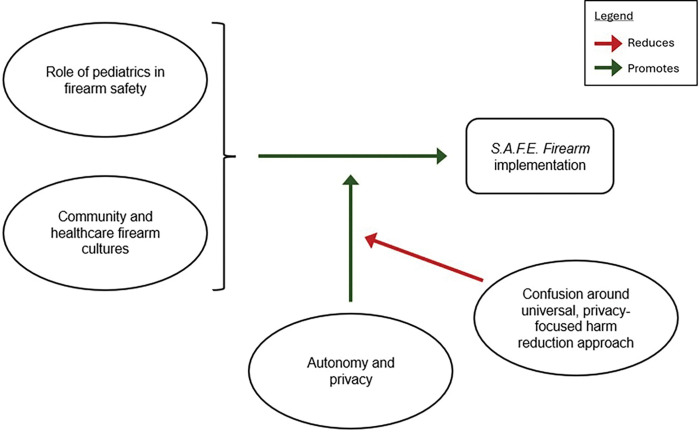
Relationships between Themes

Participants’ shared perception of the *role of pediatrics in firearm safety* served as a foundational condition for implementation success. Participants expressed that their belief that pediatric HCWs should take an active role in promoting firearm safety motivated them to deliver the program. This unified understanding of the role of pediatrics in firearm safety was especially meaningful given that participants described heterogeneity in views on firearm storage and ownership among patient families, local communities, and HCWs themselves (*community and healthcare firearm cultures)*. *S.A.F.E. Firearm*’s prioritization of *autonomy and privacy* allowed the program to align with participant beliefs about the role of pediatrics in firearm safety across these diverse community and healthcare firearm cultures. Specifically, participants highlighted that *S.A.F.E. Firearm*’s universal harm reduction approach promoted program delivery by preserving families’ autonomy and privacy regarding firearms, which were central to the acceptability of the program across these heterogeneous community and HCW firearm cultures. Finally, this substantial impact of autonomy and privacy on implementation success hinged upon participants’ full understanding versus confusion around *S.A.F.E. Firearm’s universal, privacy-focused harm reduction approach*. Streamlining with existing clinic processes and leveraging collaborative learning and reflection served as important processes affecting confusion around this approach and consequent delivery. Relationships between themes and *S.A.F.E. Firearm* delivery are illustrated in Fig. 1.

## Discussion

The participants in our study expressed a unanimous belief in pediatric HCWs’ responsibility to promote firearm safety. Corroborating these qualitative findings, in previous research, the overwhelming majority (95%) of AAP members endorsed that pediatricians should provide firearm safety counseling to patients (24). The unified commitment to firearm safety we heard from participants illustrates that many HCWs are seeking ways to support firearm safety in pediatric settings–implementation of evidence-based programs like *S.A.F.E. Firearm* represents a prime opportunity to channel this support. Health systems can effectively harness pediatric HCWs’ desire to support firearm safety through programs that have a limited burden on clinicians and emphasize HCWs’ shared commitment. Specifically, health systems can leverage brief (e.g., *S.A.F.E. Firearm*’s < 1-minute duration) programs that can be readily integrated with clinicians’ existing workflows, such as other prevention-oriented discussions (e.g., around sunscreen, seatbelts). Further, health systems should ensure HCWs understand the crux of the programs they are implementing. Based on participant input, health systems can most effectively facilitate HCW understanding of key aspects of programs like *S.A.F.E. Firearm* through targeted, succinct trainings that prioritize conveying the practical “bottom line” of program delivery to prevent confusion. By centering pediatric HCWs’ shared role in firearm safety as a public health concern, both in these trainings and in approach to program roll-out, healthcare systems can foster peer support between HCWs that can further promote program understanding and delivery.

*S.A.F.E. Firearm*’s success relied on its acceptability in the clinics and communities of implementation. Clinics, communities, and HCWs involved in this implementation initiative held heterogeneous beliefs around firearms, requiring the program to be inclusive. To effectively implement programs like *S.A.F.E. Firearm*, health systems and researchers must take into account the contexts in which they will be implemented and make adaptations to ensure their appropriateness, especially with regard to pre-existing firearm ownership rates, firearm storage practice norms, reasons for firearm ownership, and perspectives on firearm discussions in healthcare. By collaborating with clinics and HCWs as the experts on the communities they serve, and by seeking input from recipients, health systems and researchers can anticipate barriers to program acceptability and delivery and mitigate these barriers through adaptation and piloting (9, 11–13).

The program’s respect for recipient autonomy and privacy around firearms substantially enhanced its acceptability for families and HCWs alike, which in turn promoted its delivery. As one effective route for preserving recipients’ autonomy and agency, we offer *S.A.F.E. Firearm*’s universal harm reduction approach. *S.A.F.E. Firearm* is distinct from the majority of current firearm safety programs due to its lack of screening for recipient firearm ownership or storage practices (25). From the perspectives of participants, this distinction greatly contributed to preserving recipient autonomy and privacy, and therefore, the program’s acceptability. Health systems can leverage a similar approach by delivering firearm safety programs in a nonjudgemental manner and irrespective of recipient firearm ownership and storage practices. Alternative program approaches, especially those that do leverage firearm ownership or firearm storage screening, should prioritize adaptations that promote autonomy and privacy in other ways, based on recipient and HCW input. For example, our previous work has illustrated that firearm safety programs can show respect for recipient autonomy, and increase program acceptability accordingly, by emphasizing recipients’ agency regarding firearm storage decisions, adapting program names (e.g., using safety-oriented language; avoiding terms like “check,” which can connote overseeing patient behavior) and offering tangible resources that show a commitment to supporting recipients, including firearm owners (e.g., firearm locks) (9, 11, 12).

### Limitations

The primary limitations of the present study center around our interview sample. First, this analysis rests on input from HCWs; future work may benefit from including program recipients. However, our foundational work adapting *S.A.F.E. Firearm* leveraged patient family perspectives to improve the program’s acceptability, and those findings are largely consistent with the present analyses (9,11–13). Second, participants were from large healthcare systems, and their perspectives may differ from those implementing firearm safety programs in health systems with fewer resources and/or safety net settings (e.g., Federally Qualified Health Centers) or serving primarily rural or tribal patient populations. Lastly, future analyses of firearm secure storage program implementation should strive for more representative samples that include more perspectives from men, Latino/a/x individuals, and people of varying racial backgrounds.

## Conclusions

HCWs involved in *S.A.F.E. Firearm* implementation unanimously expressed that pediatric HCWs have a responsibility to promote firearm safety, consistent with previous research (24). Health systems can leverage pediatric HCWs’ commitment to firearm safety by deploying firearm safety programs that limit burden on clinicians while centering this shared belief. We recommend health systems provide brief, simple trainings that clearly convey the “bottom line” of program delivery and emphasize HCWs’ shared role in firearm safety as a public health concern. Integral to *S.A.F.E. Firearm*’s success was its appropriateness for the involved HCWs and families, who held a range of beliefs about firearm ownership and storage. Specifically, *S.A.F.E. Firearm*’s universal harm reduction approach promoted acceptability and delivery by prioritizing recipient autonomy and privacy regarding firearms, which was inclusive of this heterogeneity in firearm beliefs in the clinics and communities of implementation. To maximize impact, we recommend health systems collaborate with HCWs, clinics, and patients to adapt firearm safety programs and their implementation to meet HCW and community needs, especially by preserving recipient autonomy and privacy.

## Supplementary Material

Supplementary Files

This is a list of supplementary files associated with this preprint. Click to download.

• Supplement.docx

## Figures and Tables

**Table 1:** Demographics of Interviewed Healthcare Workers

Interviewed Healthcare Workers (N = 38)	
Gendern (%)	
Female	29 (76.32%)
Male	8 (21.05%)
Prefer not to respond	1 (2.63%)
Race	
Black or African American	3 (7.89%)
White	24 (63.16%)
Asian	3 (7.89%)
Other	3 (7.89%)
Prefer not to respond	5 (13.16%)
Ethnicity	
Hispanic or Latino/a/x	3 (7.89%)
Non-Hispanic or Latino/a/x	33 (86.84%)
Prefer not to respond	2 (5.26%)

**Table 2:** Participant Perspectives

Theme	Representative quotes
Role of pediatrics in firearm safety	A04 Clinician: “Having a staff that understands the importance of [firearm safety] is helpful…We [pediatricians] are probably ideal people to help with this because we have regular interaction with young patients and their families…because firearms are now one of the leading cause of death in children, we have an important role in counseling families on trying to prevent those deaths, if we can, through safe storage.” A09 Clinician: *“[S.A.F.E. Firearm* is] an important part [of the well-child visit]…just like we counsel about their other sources of safety, sleep and bike and car and all of this. It’s part of safety counseling. It fits in perfectly.” B07 Clinician: “Pediatricians, part of our role is to talk about safety. That’s something that we talk about starting like at the four-month visit when we tell parents, ‘Don’t leave your baby up on the bed by themselves because they’re going to start to learn how to roll.’ So we talk about safety at every single wellness visit with families…talking about gun safety is sort of a very natural and important role for us.” B17 Clinic Change Agent: “Making sure that people are aware of what they have, where they have it, how to properly store things is important for their own safety, for their children’s safety, everyone’s. Because even as a responsible gun owner, other people can come in and that can change. And so I think it’s just good to always be mindful that if you’re going to have [firearms], it comes with a level of responsibility…By mentioning it in the healthcare setting, it kind of will make people stop and think about the responsibility that comes with their firearms…any conversation that can be had to help promote that I think is great.”
Community and healthcare firearm cultures	*Community firearm cultures* B04 Clinician: “It’s pretty divalent. There’s very different opinions. There are some families that raise their kids shooting and hunting, and that is very normal for them. And there are some people who would not let their child see a gun.” A07 Clinician: “I think [firearm ownership, storage] varies a lot from patient to patient…It’s like, ‘We wouldn’t have them in the home,’ or ‘They’re definitely secured.’ But then…I know in the community that there’s a lot of firearms left around because of what I hear on the news.” A05 Clinic Change Agent: “For the most part, a good number [of my patients] have guns. And some of them admit to it, and some of them don’t. Or some of them are…kind of excited about it or they’re kind of taken aback by it too. So I definitely say a good amount have taken them so I assume that they have [guns].” A02 Clinic Change Agent: “I would say for the most part, for our demographic area and population, firearms are widely used. And so we do–our providers do a very thorough explanation of safety and how to properly store firearms and things like that just around safety in the home.” *Personal experiences with firearm violence* B05 Clinic Change Agent: “Our clinic just in the last two days was affected by a firearm-related death in someone’s immediate family…I think [firearm safety] should be the conversation that we have with everyone as part of their normal physical.” A06 Clinician: “I believe [firearm safety is] absolutely essential. Our clinic has lost several patients to firearm-related injuries…I think it’s our role and our responsibility to keep them as safe as possible.” A01 Clinician: “It’s a great idea, especially with childhood deaths from gun-related injuries being, I think, now the number one cause of death for children. I think it’s a really important thing for us to talk with our patients about. And I’ve been surprised at how many of my patients have guns and…it seems like everybody who has a gun would love a gun lock…In [local state], we had the school shooting. I think it was last year. And I think everybody in our community was rattled…it…really hit home that, ‘Okay. One of your patients might be thinking about this.’ I think that…made it feel all that more important to me.” B05 Clinic Change Agent: “I think [firearm safety is] huge. I think we, personally, in this clinic have had–we’ve seen gun violence…it’s important to have the conversations at an early age…there’s a misunderstanding that…’I grew up around guns, and my kids are going to grow up around guns. As long as I teach them responsibility, it’s fine.’ And so I think having the conversation from a pediatrician or from a care provider that’s trusted to say, ‘Hey, I hear that, and I’m not invalidating your views, but also, what happens when other kiddos come over that don’t have that background?’” *Perspectives of healthcare workers with a history of firearm ownership* A06 Clinician: “In terms of the culture, many of my parents either are police officers or believe in carrying weapons. So because that is the case, I determined a long time ago not to really counsel against having firearms. We also have a lot of veterans, and my father is Southern, and I grew up with guns in my home. So I don’t share the traditional pediatric belief that we should never have anything in our house. But what I do believe is that we need to show people how to be safe.” B04 Clinician: “I think it’s easier for me than some people, in a way, because I can see both sides of it right now. I grew up in a house where there were guns, and then I will tell people, ‘Yeah, I don’t know. I grew up and my dad had a couple of rifles in the downstairs closet, and the bullets were like 30 feet away in the kitchen in a drawer. And so yes, I understand that your kid is being raised to not touch these things and to be responsible with them. That’s how I was raised as well. But statistics are still statistics, and we’re doing this, the same as wearing helmets, to try and reduce the number of accidents and bad things happening. And ultimately, these questions are all about keeping kids alive, so.’ And it gives a little bit more credibility to it as opposed to, ‘No, I’ve never seen a gun. Guns are icky, and they’re the worst thing ever. They should all be burned.’ And so I think being able to talk to people in a way that makes it feel less judgmental because there’s at least some shared commonality to it probably helps.”
Autonomy and privacy	*“Polarizing” topics in healthcare* B20 Clinician: “As the program was initially rolling out, I think all of the providers in our clinic, myself included, were feeling really apprehensive about discussing firearm storage with families. And I think a lot of that came from a perception that it’s a polarizing topic and that it might be something that would put people on their guard or that they might not feel comfortable talking about with their doctor. And I think we felt like there were so many other topics like that already, like COVID vaccines and everything else, that we were having to talk with families about that I think we’re all feeling kind of apprehensive about how this would go…pretty quickly, we had a lot of really positive feedback from our providers on how it went…a lot of my patients who do own firearms were actually happy that I asked or grateful that it’s something that we’re talking about” B08 Clinician: “There’s that kind of political and cultural divide in our country that I think certainly has an influence in any of this kind of discussion. People may be more emboldened to say no and maybe vigorously say no.” S.A.F.E. Firearm *versus screening-based firearm safety programs* A01 Clinician: “I tried to present it in a neutral way because…I didn’t want people who have a firearm to feel like I was judging them for having one. If they did want the gun lock, I want it to be like, ‘This isn’t a big deal. We do this for everybody’…I try not to approach it differently for some families rather than others.” A03 Leader: “In my experience, people can get very defensive, again, thinking [you’re] asking because you’re anti-firearm, but that’s not the case. [We] just want people to be safe and understand the dangers. So I think giving people options and, particularly, if nothing else, giving them safe storage ideas, is a terrific idea.” B01 Clinician: “In the past, when we have talked about firearms, the conversation…was just a little trickier in terms of not wanting people to feel judged and people potentially getting a bit more defensive. And I have to say that since we have started this project where we actually have the cable locks to offer and talk about why we’re doing it, those conversations, I think, have actually gone quite a bit better. And I almost find that the gun owners are even a little bit more engaged, certainly than they were for conversations previously. But they really actually seem appreciative that we’re doing it, and it comes off in a less judgmental way…when they used to fill out those surveys, I don’t think everybody answers honestly…[now] I say, ‘Hey, we’re offering these locks to gun owners and potentially non-gun owners alike in case anybody is visiting that might need to secure a firearm, or you guys go anywhere else that you feel like a firearm might need to be secured.’ So that just kind of covers all the bases. And it’s a little more disarming than making people feel like, ‘Well, I didn’t answer that I had a gun.’” *Exchanges between healthcare workers with varied firearm familiarity* A06 Clinician: “As I teach my [medical] students or speak to my colleagues, I grew up in a home where there were loaded firearms within reach, and we just didn’t touch them. That’s just kind of how it was…now we can’t really assume that that’s the case.” B22 Clinician: “A patient once asked me how the cable lock worked. And being that I don’t own guns, I’ve never really even touched a gun, and I don’t understand how they work, I took it to a coworker who walked me through it.” A02 Clinic Change Agent: “My only difficulty is that I am not familiar with firearms very much in my personal life. So even the locks were new to me. But again, it’s all a learning process, especially with the demographic area of our clinic…I did a lot of self-learning. I asked around with the staff.”
Confusion around *S.A.F.E. Firearm’s* universal, privacy-focused harm reduction approach	*Healthcare worker confusion:* B06 Clinic Change Agent: “Our questionnaire, it’s like, ‘Do they have a gun or not in the house? Is it safely stored?’ I know that’s separate…from your study…that’s in our general questionnaires…[but] I had some providers come to me, ‘Why don’t they, say, put in [the electronic health record] that we talked about [how] they have a gun.’” A08 Clinician: “I have a few parents that freak out right away when I say, ‘Do you have firearms in the house?’” B10 Clinician: “50% of the time, I don’t even have to talk about it because they don’t have guns.” A11 Clinician: “If the firearm is absent, I personally don’t give a cable lock…it would be nicer if there [were] an embedded [electronic health record] option saying, ‘No gun in home. Cable lock not offered.’” *Strategies for reducing and preventing confusion:* B02 Leader: “It’s been helpful that we’ve been able to have some department meetings and trainings…that upfront training…looking at different videos and ways of delivering the counseling, I think that was really helpful…We had an initial discussion about sort of what the workflow would be, and if families asked questions, how we would handle that…[Facilitator] had shared some data, how we were doing as providers with the counseling, so that was helpful.” A07 Clinician: “I thought [the trainings] were great…Yes, I did [feel ready to deliver *S.A.F.E. Firearm*]…[The terminology] probably was the most helpful thing because prior to that, it was our habit to ask, ‘Do you have firearms at home?’ And you may not get an honest answer with that. So I think it was very beneficial to have the wording be, ‘If there are firearms, this is what we recommend.’ And I think people hear that better than having to answer the question. I’m not sure we would always get an honest answer.” Interviewer: “What changes do you think we need to make to the program to make it better?” B23 Leader: “More continued kind of interaction with providers, continuing to offer more educational tools, more kind of peer-to-peer engagement, meaning if this doc over here has done a really nice job with the counseling or they’re showing a lot of success…more of a regular communication forum or an open forum for clinicians to come to.” B20 Clinician: “We also set up some time in our provider meetings to talk about the *S.A.F.E. Firearm* program multiple times throughout the study. At the beginning, it was to talk about implementation and to review some of the training videos from the [American Academy of Pediatrics] on discussing safe firearm storage with families…And then it was touching base with our team to share updates that we were getting about our data, to troubleshoot problems that people were having, and get feedback on their experiences…One thing that I was really grateful about with the implementation of the program was that there was a lot of support for us on providing some education on how to do this, because I think it was not really part of any of our practices at the time that the program was implemented. I don’t think anybody was really asking routinely about it, although it was a question that was on our questionnaires. And so in particular, it was really helpful to have the videos to review to watch somebody do this, I guess, or to see what some good verbiage might be…” A14 Clinician: “We did meetings…[on] how are we going to implement [the electronic health record nudge], what needed to be done to get it going in our office…I felt like it was helpful, especially because we’re a training facility, to have it actually written out like, ‘If there’s a firearm in the house, we want to make sure it’s safely stored and kids can’t have access to it.’ So in some of my [medical] student notes, I kind of put that in there to kind of give them a prompt because we’re not asking if [patients] have a firearm… It’s educational for a lot of our trainees that we’re not actually asking if [patient families] have a firearm. We don’t care if they have a firearm. We just want to make sure that if there’s a firearm, that it’s safely stored, and then we want to offer them the cable locks.”

## Data Availability

Due to ethical and confidentiality concerns, qualitative data reported on in this manuscript will not be made available. Previously reported quantitative data referred to in this manuscript are currently available and can be access in the National Institute of Mental Health (NIMH) Data Archive at https://nda.nih.gov/data-structure/aspire_ehr01. This includes deidentified individual participant data and data dictionaries. These can be accessed by those who meet NIMH Data Archive data access requirements and receive NIMH Data Archive Data Access Committee approval. Analysis proposals should be submitted to the NIMH Data Archive Data Access Committee, which can be contacted via email at ndahelp@mail.nih.gov. Additional study documents relevant to our qualitative analysis are included in the supplement of the present paper (i.e., qualitative interview guide).

## Abbreviations

S.A.F.E.: (*Suicide and Accident Prevention through Family Education*) Firearm
HCW: healthcare worker
ASPIRE: Adolescent and child Suicide Prevention in Routine clinical Encounters
